# Long-Term Management of Generalised Anxiety Disorder with Low-Dose Continuous Infusions of Flumazenil: A Case Series

**DOI:** 10.3390/bs12110430

**Published:** 2022-11-02

**Authors:** Alexander T. Gallo, Stephen Addis, Vlad Martyn, Hishani Ramanathan, Grace K. Wilkerson, Sean D. Hood, Hans Stampfer, Gary K. Hulse

**Affiliations:** 1 Division of Psychiatry, Medical School, The University of Western Australia, Nedlands, WA 6009, Australia; 2 Fresh Start Recovery Programme, Subiaco, Western Australia, Subiaco, WA 6008, Australia; 3 School of Medical and Health Sciences, Edith Cowan University, Joondalup, WA 6027, Australia

**Keywords:** GABA, anxiety disorder, treatment response, flumazenil, infusion, remission

## Abstract

Background: Generalised anxiety disorder (GAD) is a common anxiety disorder associated with social and occupational impairment. Recently, a theory was postulated that dysfunctional gamma aminobutyric acid type A receptors (GABA_A_) are implicated in anxiety symptomology, which could be corrected by flumazenil, an antagonist at the benzodiazepine binding site on the GABA_A_ receptor. Method: Participants had a primary diagnosis of GAD and were treated initially with an eight-day continuous low-dose flumazenil infusion (total 32 mg at a rate of 4 mg/24 h). Some participants were re-treated with a further four- or eight-day infusion. Treatment response was measured as a 50% reduction in anxiety or stress scores on the Depression Anxiety Stress Scale—21 (DASS-21). Remission was measured as scores ≤3 or ≤7 on the anxiety and stress subscales of the DASS-21, respectively. Results: Eight cases are reported. All cases met the criteria for treatment response on the anxiety and stress subscale of the DASS-21. Remission was achieved in seven participants on the anxiety subscale and in five on the stress subscale. No changes in hepatic, renal, or haematological function were likely attributed to flumazenil. Conclusion: Data suggest that low-dose continuous flumazenil infusion manages GAD symptoms and is safe. Although these results are promising, future randomised control trials are required to confirm these results.

## 1. Introduction

Generalised anxiety disorder (GAD) is one of the most common anxiety disorders, which is associated with significant social and occupational impairment. The *Diagnostic and Statistical Manual for Mental Disorders Fifth Edition* (DSM-5) defines the key feature of GAD to be excessive anxiety and worry [1]. The anxiety associated with GAD in adults is typically about every day, routine life circumstances including job responsibilities, own health or the health of family members, and other minor matters [1]. The mean onset of GAD has been estimated at 21 years; however, when GAD presents as a primary disorder, the onset tends to be in the early teenage years [2]. Unfortunately, GAD is chronic and likely to be episodic or unremitting [3,4]. Accordingly, several non-pharmacological and pharmacological treatments exist. 

First-line pharmacological treatment options for GAD typically involve treatment with a selective serotonin reuptake inhibitor (SSRI) or serotonin noradrenaline reuptake inhibitor (SNRI), primarily acting on serotonin (5-HT) and/or noradrenaline transporters. More recently, there has been a move away from this typical mechanism to more novel actions. One example is agomelatine, which acts as a melatonin receptor agonist and 5-HT_2C_ antagonist [5,6]. Although agomelatine comes with its own side effects, they do not include some of the more bothersome effects associated with SSRIs and SNRIs (e.g., sexual dysfunction and psychomotor agitation) [7,8]. Additionally, agomelatine is not associated with a withdrawal syndrome like SSRIs and SNRIs [9]. Given agomelatine’s favourable side effect profile, a move away from monoaminergic drugs may provide patients and clinicians with more treatment options. This would be particularly beneficial for patients discontinuing first-line treatments due to side effects or those demonstrating treatment resistance, which is estimated to be approximately 30% of patients with anxiety disorders [10]. 

Although numerous neurotransmitters have been implicated in anxiety disorders, the focus has primarily been directed at monoamines. Gamma-amino butyric acid type A (GABA_A_) receptors have also been the target of the widely used drug class, benzodiazepines. A recent theory proposed that dysfunctional GABA_A_ receptors, resultant of chronic exposure to and subsequent withdrawal from allopregnanolone, are implicit in anxiety disorders [11]. The theory postulates that allopregnanolone, a positive allosteric modulator of the GABA_A_ receptor with an anxiolytic effect, increases α_4_βδ GABA_A_ receptor expression, causing allopregnanolone to have a paradoxical anxiogenic action [11]. Flumazenil, typically characterised as an antagonist at the benzodiazepine binding site on the GABA_A_ receptor, has been shown to remove these receptors via internalisation [12]. The removal of these receptors may, therefore, result in the typical anxiolytic response to endogenous allopregnanolone and decrease anxiety symptoms. Not unlike treatment with ketamine infusions, the effects are thought to last beyond the duration of the infusion. Theoretically, symptoms would return after exposure to chronic stress. Unfortunately, the pharmacokinetic profile of flumazenil is not conducive to oral administrated, with a short half-life, low bioavailability, and high first-pass metabolism [13]. To overcome this, flumazenil can be delivered subcutaneously through continuous low-dose infusions, as bolus doses may be anxiogenic [14,15]. 

We previously demonstrated a significant reduction in anxiety, stress, and sleep disturbance symptoms after treatment with a continuous low-dose flumazenil infusion over approximately eight days in an open label naturalistic pilot study. Significance was maintained 20 days after the infusion ended (unpublished data). Here, we report the long-term safety and efficacy results from the eight patients, which had data available for six months and beyond. 

## 2. Method

### 2.1. Design

A longitudinal case series of participants being treated with subcutaneous flumazenil infusions for GAD. 

### 2.2. Clinical Setting

Participants were under the care of a general practitioner at an outpatient class B day hospital (Subiaco, Western Australia), which is licenced to perform procedures under sedation, or that involve the invasion of a sterile body cavity [16], and were initially assessed by a psychiatrist. The study was approved by Southcity Medical Centre Human Research Ethics Committee (001//2019) and recognised by the University of Western Australia Human Research Ethics Committee (2019/RA/4/20/5926). 

### 2.3. Participants

Participants meeting the inclusion criteria were recruited consecutively after referral by a psychiatrist or psychologist to the outpatient clinic for assessment and potential treatment of GAD symptoms using flumazenil. All participants underwent an assessment by the treating psychiatrist to assess the primary diagnosis and a history of treatment resistance. Treatment resistance was defined as having received or currently receiving a therapeutic dose of any pharmacotherapy for GAD for an adequate period and still experiencing clinically significant symptoms. 

### 2.4. Inclusion and Exclusion Criteria

Inclusion criteria were: (1) adult aged 18 years and over, (2) primary diagnosis of GAD by the DSM-5 criteria, and (3) willing and able to give informed consent for data collection. Exclusion criteria were: (1) using daily benzodiazepines (self-reported) as flumazenil has been shown to affect benzodiazepine use and withdrawal [15,17], (2) initiation or dose change of any psychotropic medication that could be used in the management of GAD in the last six weeks, (3) received low-dose continuous flumazenil infusion(s) for any indication previously, (4) received sustained release flumazenil, (5) currently pregnant or breastfeeding, and (6), untreated hyperthyroidism, which may mimic anxiety symptoms. 

### 2.5. Intervention

All participants were initially treated with two consecutive subcutaneous flumazenil infusions, each containing 16 mg of flumazenil in a 30 mL solution, delivering a total dose of 32 mg. The flow rate was designed to deliver approximately 4 mg/24 h period (±20%) as described previously [14]. As such, the total duration of the two infusions was approximately eight days. Drug delivery was achieved using a syringe pump (SpringFusor^®^ 30-AB; Go Medical Industries, Subiaco Western Australia), which consists of a spring-driven cartridge to deploy the syringe and flow control tubing to deliver at a rate of 0.31 mL/h. The benefit of this pump is that it allows participants to remain ambulatory for the duration of the infusions and could be worn around the neck with a lanyard or in a belt. Participants were maintained on their current medications for GAD. Sodium valproate 500 mg was given twice a day as seizure prophylaxis for the duration of the infusion in participants that were assessed to be at risk of seizure due to chronic alcohol use. This decision was based on the documented low risk of seizures during benzodiazepine withdrawal using flumazenil [18], as benzodiazepines have a similar mechanism of action to alcohol. Participants were re-treated on an as-needed basis suggested by the treating psychiatrist. The duration of the second infusion was based on clinical need and patient preference. As such, those that were re-treated received approximately a four-day (16 mg) or eight-day (32 mg) treatment. Blood samples were taken at baseline and opportunistically throughout the follow-up period (urea and electrolytes [U&E], liver function test [LFT], full blood count [FBC]). A thyroid function test (TFT) was completed at baseline to exclude hyperthyroidism, which is a differential diagnosis for GAD. 

### 2.6. Outcome Measures

Participants completed the Depression Anxiety Stress Scale—21 (DASS-21) [19] and six-item Spielberger State Anxiety Inventory (SSAI-6) [20] at baseline and approximately days 4, 8, 14, 28 (±1 day), and monthly (±7 days) to six months. The Jenkins Sleep Scale (JSS) was measured at baseline and monthly (±7 days) to six months. The Spielberger State-Trait Anxiety Inventory—Trait (STAI-T) [21] was collected at baseline and approximately three and six months (±7 days). The primary outcome measure was time to treatment response and time to remission, measured by the anxiety and stress subscales on the DASS-21. Treatment response was defined as a reduction in stress or anxiety of 50% or more and remission was defined as an anxiety score ≤3 or a stress score ≤7, which has been described previously [22]. 

The DASS-21 is a well-established instrument for measuring depression, anxiety, and stress with good reliability and validity across a number of populations [23]. Scores reliably differentiate samples of patients with anxiety disorders from non-clinical samples [24] and the anxiety and depression subscales distinguish between patients with anxiety diagnoses and depressive diagnoses [24,25]. Frequency/severity ratings are made on a four-point scale (0 = did not apply to me at all, 3 = applied to me very much, or most of the time) based on the past seven days, with a total score range of 0–21 on each subscale. Higher scores indicate higher levels of depression, anxiety, or stress. 

The SSAI-6 has acceptable reliability and validity, producing scores similar to those obtained from the full 20-item Spielberger State Anxiety Inventory in several groups with varying degrees of anxiety [19]. It is sensitive to fluctuations in state anxiety and offers a briefer measure for participants’ convenience. Ratings are made on a four-point scale (1 = not at all, 4 = very much so) based on how they feel right now, with higher scores indicating higher levels of state anxiety. 

The JSS is a brief sleep questionnaire for research, which evaluates the frequency in the last month of difficulties falling asleep, waking during the night, trouble remaining asleep, and subjective feelings of fatigue and sleepiness after normal sleep [26,27]. Ratings are made on a five-point scale (0 = not at all, 5 = 22–30 days) and it has been shown to have good predictive validity [27]. 

The STAI-T evaluates trait anxiety, which is the disposition to be anxious in perceived threatening situations. It comprises of 20-items and is rated on a four-point scale (1 = not at all, 4 = very much so) based on how they generally feel, with higher scores indicating higher trait anxiety [21]. The scale has high discriminant and convergent validity with other measures of anxiety [21]. 

## 3. Case Reports

### 3.1. Participant Characteristics

The participant characteristics are summarised in Table 1.

### 3.2. Cases

**Figure 1:**
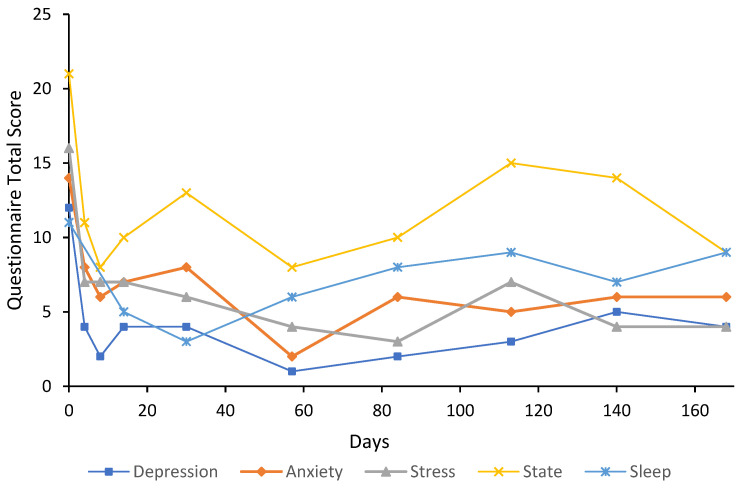
Line graph showing Case 1 results from the DASS-21 (depression, anxiety, and stress subscales), SSAI-6, and JSS at each follow-up. The participant was treated with an eight-day flumazenil infusion without re-treatment during the follow-up period.

***Case 1*** was a 29-year-old female who had a history of GAD with treatment resistance and major depressive disorder (MDD) for 10 years. The participant had previously taken escitalopram for over a year; however, there was little effect on anxiety symptoms, and this treatment was ceased due to lack of efficacy and side effects. The participant started 25 mg of agomelatine, which had a “moderate” effect on depressive symptoms, although anxiety symptoms persisted. In addition to this, the participant had taken diazepam and alprazolam when required for anxiety. The participant received psychotherapy intermittently since she was approximately 15 years old. The participant reported some recreational drug use as a teenager and in their early 20s (3,4-methylenedioxymethamphetamine, cannabis, and alcohol); however, this never developed into a substance use disorder. At the time of presentation, the participant was under the care of a psychiatrist with a provisional diagnosis of attention-deficit/hyperactivity disorder (ADHD) and taking lisdexamfetamine, which may affect sleep results. U&E, LFT, FBC, and TFT were unremarkable at baseline. The participant received an eight-day flumazenil infusion. Measures of anxiety at baseline were very severe (14) for anxiety and severe for stress (16) on the DASS-21; state anxiety was also high at 21 out of a possible 24 (Figure 1). Treatment response was achieved by day eight for anxiety and maintained for the six-month follow-up period except for the day 28 follow-up, where the participant scored eight on the anxiety subscale. Treatment response was achieved on the stress subscale by day four and maintained for the six-month follow-up period; remission was also achieved on this subscale for the entire follow-up period. State anxiety, depression subscale on the DASS-21, and sleep scores also decreased from baseline and remained below baseline for the six-month follow-up period. On day 468, depression, anxiety, and stress subscale scores were 3, 2, and 11, respectively, indicating maintenance of remission of anxiety symptoms. State anxiety was 13 and the sleep score was eight. At this time, psychiatric medications were slow-release dexamfetamine (25 mg/day) for ADHD; lisdexamfetamine and agomelatine had been ceased. Trait anxiety at baseline was 69, which had decreased to 45 by day 84 and slightly increased to 54 by day 168. At day 468, trait anxiety had decreased to 46. U&E, LFT, and FBC results remained unremarkable on days 8, 33, and 90. Unfortunately, benzodiazepine use over this period was not recorded.

**Figure 2:**
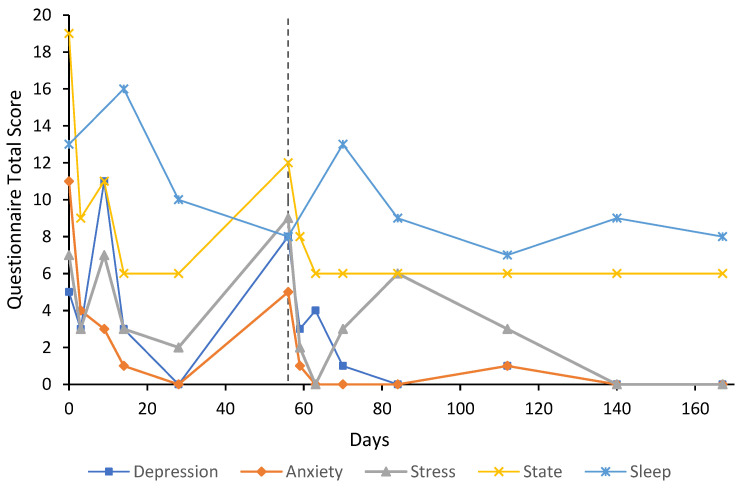
Line graph showing Case 2 results from the DASS-21 (depression, anxiety, and stress subscales), SSAI-6, and JSS at each follow-up. The participant was treated with an eight-day flumazenil infusion and re-treated on day 56 with a four-day flumazenil infusion (denoted by dashed line).

***Case 2*** was a 35-year-old female, mother of three, with a 15-year history of GAD with treatment resistance and more recent alcohol abuse, to “cope with stress”. The participant was taking 20 mg of escitalopram at baseline and took 0.25 mg of alprazolam and 25 mg of quetiapine occasionally. The escitalopram dose had been increased from 10 mg approximately six months before the baseline assessment with no change in symptoms. The participant was under the care of a psychiatrist upon presentation and attended psychotherapy with a psychologist during the follow-up period. Anxiety and stress subscale scores were extremely severe and normal, respectively, at baseline; U&E and FBC were unremarkable, LFT showed slight elevations in aspartate aminotransferase (AST) and gamma glutamyl transferase (GGT) at 33 U/L and 49 U/L, respectively. The participant initially received an eight-day flumazenil infusion plus 500 mg of sodium valproate twice daily for seizure prophylaxis due to a history of alcohol abuse. The participant showed treatment response on anxiety and stress subscale scores by day four, which was maintained for anxiety for the duration of the six-month follow-up period (Figure 2). The stress score remained in the normal range until day 56, when it had increased to mild (9). At day 56, the participant was re-treated with a four-day flumazenil infusion (total of 16 mg), which seemed to manage anxiety symptoms again. From here, stress scores remained below the baseline until the six-month follow-up. After approximately four weeks from baseline, the participant felt ready to come off escitalopram as it was not efficacious and she “did not like the way it made [her] feel”. Although the participant was advised to taper the dose slowly, they stopped after approximately one to two weeks and did not report any significant withdrawal effects. Depression subscale, state anxiety, and sleep scores fluctuated over the six-month follow-up period and were mostly lower than baseline scores. The participant reported that the treatment had “saved [her] life”. At day 423, depression, anxiety, and stress scores were 0, 2, and 3, respectively, which were all lower than baseline. State anxiety and sleep score were 8 and 12, respectively. State anxiety was 11 points below baseline while sleep had almost returned to the baseline level of 13. The participant was not taking any psychiatric medication at this follow-up. Trait anxiety at baseline was 56, which had decreased to 37 by day 84 and further decreased to 25 by day 167. On day 423, trait anxiety was 32. At day 59, during the second flumazenil treatment, U&E was unremarkable, LFT showed further elevations in AST (45 U/L), GGT (86 U/L), and alanine aminotransferase (ALT, 39 U/L); however, this is likely attributed to alcohol consumption. Borderline macrocytosis was noted on the FBC, which was still observed at day 81; LFT had returned to normal. On day 167, all measures on U&E, LFT, and FBC were within normal range. Unfortunately, benzodiazepine use over this period was not recorded.

**Figure 3:**
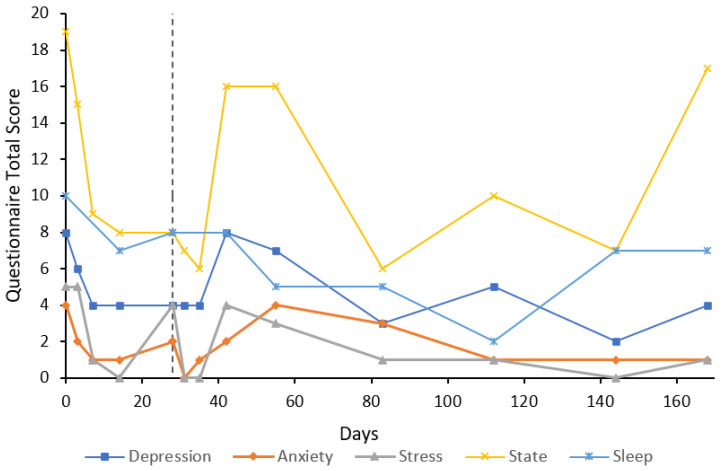
Line graph showing Case 3 results from the DASS-21 (depression, anxiety, and stress subscales), SSAI-6, and JSS at each follow-up. The participant was treated with an eight-day flumazenil infusion and re-treated on day 28 with a four-day flumazenil infusion (denoted by a dashed line).

***Case 3*** was a 64-year-old male, retired paramedic, with a 15-year history of GAD with treatment resistance. The participant also had a historic diagnosis of posttraumatic stress disorder with persistent residual symptoms. Previously, the participant had trialled agomelatine and amitriptyline; however, both were ineffective, and amitriptyline was ceased due to mood side effects of increased anxiety, anger, and irritability. Sleep was disturbed; the participant was waking nightly around 3 a.m. with anxiety and was unable to go back to sleep. Temazepam was briefly trialled approximately five months prior to the baseline assessment but the participant was reluctant to continue with this. The participant was taking CoQ10, vitamin D3, magnesium, zinc, vitamin B12, and ashwagandha. Baseline anxiety and stress scores were low at baseline, despite describing significant anxiety symptoms. Anxiety and stress subscale scores were mild (4) and normal (5), respectively (Figure 3). The participant continued to receive psychotherapy from the treating psychiatrist (S.A.) and engaged in Christian-based counselling. U&E, LFT, FBC, and TFT were all unremarkable at baseline. Treatment response was achieved on the anxiety subscale by day four and was maintained at all follow-ups except for the two- (4) and three-month (3) follow-ups; however, the participant had been stressed about planning a family wedding, which may account for this. Treatment response was achieved on the stress subscale at most follow-ups. Anxiety and stress levels never exceeded baseline levels. The participant felt they had “lower baseline anxiety levels” from the first eight-day infusion, and at their request, received a further four-day infusion (16 mg) at day 28, which again reduced subjective baseline anxiety levels. Remission was achieved for most of the follow-up period for anxiety. Remission on the stress scale was not assessed as their baseline score was already in the normal range. State anxiety and sleep scores remained below baseline for the duration of the follow-up period. The participant had a myocardial infarction at approximately the five-month follow-up. At day 394, depression, anxiety, and stress scores were 4, 0, and 2, respectively, which were lower than baseline scores. State anxiety was 9 compared to 19 at baseline and sleep score was 7 compared to 10 at baseline. The participant was taking no new psychiatric medications. Trait anxiety at baseline was 59 and had decreased considerably to 29 on day 83. By day 168 it had increased to 48, which may be attributed to the stress from the myocardial infarction. By day 394, trait anxiety had decreased again to 31. There were no remarkable changes in U&E, LFT, and FBC results on days 8, 29 (one day after the second treatment began), and 99. 

**Figure 4:**
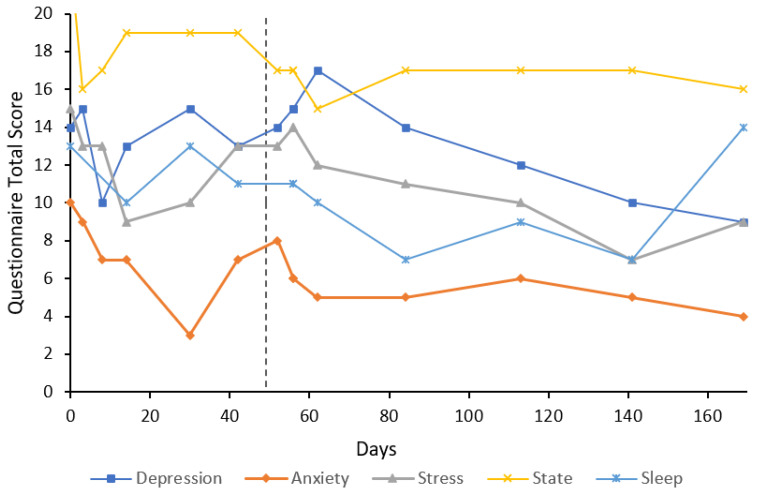
Line graph showing Case 4 results from the DASS-21 (depression, anxiety, and stress subscales), SSAI-6, and JSS at each follow-up. The participant was treated with an eight-day flumazenil infusion and re-treated on day 49 with a four-day flumazenil infusion (denoted by a dashed line).

***Case 4*** was a 51-year-old female participant with a 20-year history of GAD with treatment resistance and comorbid social anxiety disorder who had trialled several SSRIs and SNRIs with limited benefit. At the time of the presentation, the participant was taking progesterone for endometriosis but was taking no psychiatric medications. U&E, LFT, FBC, and TFT were unremarkable at baseline. Baseline anxiety (10) and stress (15) subscale scores were extremely severe and severe, respectively (Figure 4). Treatment response and remission were achieved by day 30 on the anxiety subscale (3) and by day 141 on the stress subscale; however, this was not always maintained. On day 42, the participant was re-treated with an eight-day flumazenil infusion due to the return of anxiety symptoms. The depression subscale scores on the DASS-21 fluctuated above and below the baseline score of 14, with a maximum score of 17, representing extremely severe depressive symptoms, despite never receiving a diagnosis of MDD. State anxiety scores also remained high, ranging from 15–23 during the six-month follow-up period. Sleep scores ranged from 7–13. At day 345, depression, anxiety, and stress scores were moderate (8), mild (4), and moderate (10), respectively; state anxiety was at its lowest (12) and the sleep score was 8. The participant had not received any psychotherapy or taken any psychiatric medication from baseline to day 345. Trait anxiety at baseline was 65, which had decreased to 52 by day 84. On day 169, trait anxiety was 49 and on day 345 had increased to 56. U&E, LFT, and FBC remained unremarkable on day 42 and day 56 (end of re-treatment period).

**Figure 5:**
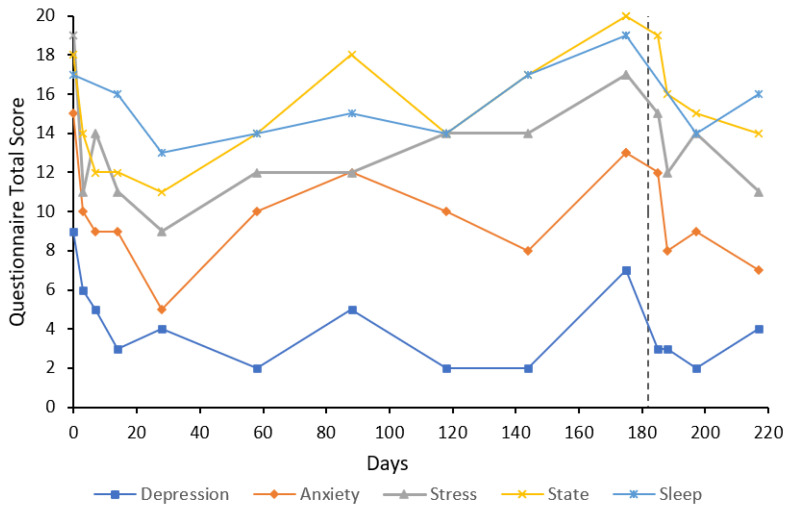
Line graph showing Case 5 results from the DASS-21 (depression, anxiety, and stress subscales), SSAI-6, and JSS at each follow-up. The participant was treated with an eight-day flumazenil infusion and re-treated on day 182 with an eight-day flumazenil infusion (denoted by a dashed line).

***Case 5*** was a 20-year-old female, undergraduate university student, with an eight-year history of GAD with treatment resistance and comorbid social anxiety disorder. The participant had previously taken paroxetine and fluoxetine with no effect on anxiety symptoms. Poor sleep quality was reported, waking multiple times a night with nightmares about a previous abusive relationship and bullying. U&E, LFT, and TFT were unremarkable at baseline. Baseline anxiety and stress subscale scores were both extremely severe at 15 and 19, respectively (Figure 5). Treatment response was achieved by day 28 on both subscales; however, remission was never achieved in this participant. Increases in anxiety measures at day 88 were associated with significant stress due to an accident involving a close family member. Re-treatment was offered prior to this at approximately 90 days; however, given the circumstances and a needle phobia, the participant declined. The participant was re-treated with an eight-day flumazenil infusion at day 182 due to worsening anxiety symptoms, which the participant reported had improved since the first infusion. Following this, treatment response was achieved at day 217, which was 35 days after the second eight-day infusion began. State anxiety fluctuated above and below baseline (18) and ranged from 11–20. The participant reported some improvements in sleep occurring shortly after the flumazenil infusions; sleep scores ranged from 13–19 with a baseline score of 17. Trait anxiety at baseline was 57 and was essentially unchanged at day 88 at 56. By day 175, trait anxiety had increased to 66; however, 35 days after re-treatment, trait anxiety had decreased to 48. No further blood tests were completed due to the needle phobia. 

**Figure 6:**
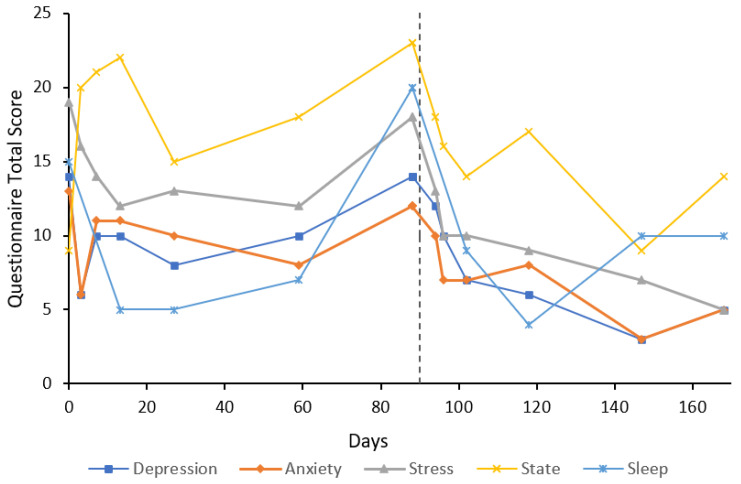
Line graph showing Case 6 results from the DASS-21 (depression, anxiety, and stress subscales), SSAI-6, and JSS at each follow-up. The participant was treated with an eight-day flumazenil infusion and re-treated on day 90 with an eight-day flumazenil infusion (denoted by a dashed line).

***Case 6*** was a 54-year-old male, formwork constructor, with a five-year history of GAD with treatment resistance. The participant also had a history of cannabis, amphetamine, and alcohol abuse through late high school; however, only alcohol use has remained an intermittent issue. At the time of presentation, psychiatric medications were 20 mg of fluoxetine and 150 mg of slow-release bupropion, which was changed to 90 mg/8 mg of bupropion/naltrexone twice a day approximately three months after baseline. U&E, FBC, and TFT were unremarkable at baseline. ALT was slightly elevated (52 U/L), which may be attributed to alcohol consumption. The participant started psychotherapy on day 115 and had been receiving alcohol cessation advice. Baseline anxiety and stress scores were both extremely severe at 13 and 19, respectively (Figure 6). The participant received an initial eight-day flumazenil infusion; however, they were under significant stress due to the sudden death of their father. The participant felt that they had an “unfair” result from flumazenil and were treated again with another eight-day infusion on day 90. Treatment response and remission on the anxiety subscale were achieved by day 147; however, only the treatment response was sustained at the final follow-up. Treatment response was achieved on the stress subscale at day 118, which was 28 days after re-treatment was commenced; this response was sustained through to day 168 (final follow-up). Remission on the stress subscale was achieved by day 147 and was sustained at the final follow-up. State anxiety was relatively low at baseline with a score of 9 and did not decrease below this throughout the six-month follow-up period. Sleep scores were improved from baseline (15) at day 27 (5) and day 59 (7) but had increased by day 88 (20). After re-treatment, sleep improved by day 112 (4) and remained at 10 until the end of the follow-up period. It is important to note that the participant had a provisional diagnosis of obstructive sleep apnoea at baseline, which may have affected these results. At day 391, depression, anxiety, and stress were 5, 7, and 10, respectively; the state anxiety score was 18, and the sleep score was 12. Trait anxiety at baseline was 66 and had increased to 73 by day 88, which may be attributed to the loss of a close family member; however, on day 168 and after a second eight-day treatment, trait anxiety had decreased to 51. Trait anxiety had increased to 59 by day 391. At day 108, U&E and FBC were unremarkable; however, AST, ALT, and GGT were all elevated (36 U/L, 47 U/L, and 55 U/L, respectively). This may be attributed to alcohol consumption or the introduction of oral naltrexone. 

**Figure 7:**
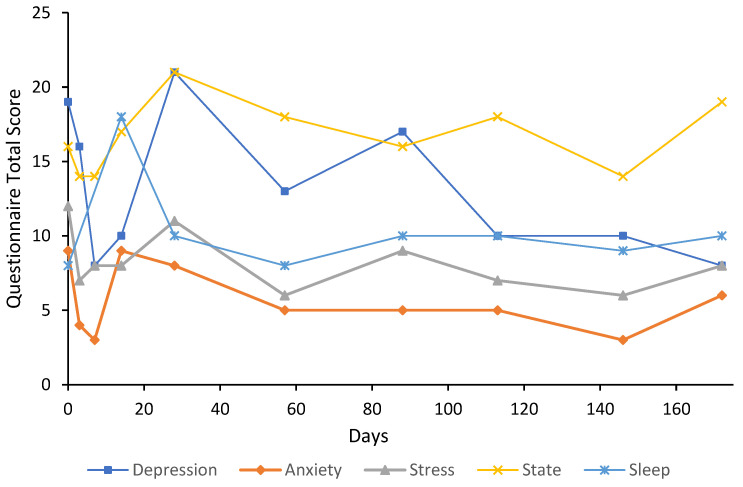
Line graph showing Case 7 results from the DASS-21 (depression, anxiety, and stress subscales), SSAI-6, and JSS at each follow-up. The participant was treated with an eight-day flumazenil infusion without re-treatment during the follow-up period.

***Case 7*** was a 31-year-old male with a 20-year history of GAD and comorbid MDD. The participant had previously taken mirtazapine for MDD, which was not given an adequate trial as it did not “agree with [him] at all”. Accordingly, the participant was reluctant to try other antidepressant therapies. At baseline, anxiety and stress subscale scores were severe (9) and moderate (12), respectively. The depression subscale score in this participant was considerably higher than the other participants at 19, which is only two points lower than the maximum score. U&E, FBC, and TFT were unremarkable at baseline. Bilirubin (25 μM/L), AST (47 U/L), and ALT (68 U/L) were elevated. The participant received an eight-day flumazenil infusion for the management of anxiety symptoms. By day four of flumazenil treatment, bilirubin (24 μM/L), AST (41 U/L), and ALT (59 U/L) had slightly decreased. No further blood tests were taken as the participant did not return to the clinic. Treatment response on the anxiety subscale was achieved by day three and remission by day eight (Figure 7). However, by day 14, the anxiety score had returned to baseline and remained somewhat steady until day 146 when it had decreased to 3 (remission), which was not sustained at the final follow-up. Treatment response on the stress subscale was achieved at day 57 and remission was achieved periodically throughout the follow-up period. Depression scores were lowest on day 7 and 172 at 8; however, they reached the maximum score of 21 by day 28 and ranged from moderate to extremely severe. State anxiety scores remained high throughout the follow-up period and sleep scores ranged from 8 at baseline to 18 on day 14. At day 345, depression, anxiety, and stress scores were moderate (8), moderate (6), and normal (7), respectively; state anxiety returned to baseline (16) and the sleep score was 9, which was slightly above baseline (8). The participant only attended two psychotherapy sessions during the initial six-month follow-up period and had not attended any additional sessions or started any medication for anxiety or depression between the six- and twelve-month follow-ups. Trait anxiety remained relatively unchanged in this participant from baseline (65) to day 88 (66), day 172 (63), and day 372 (56). 

**Figure 8:**
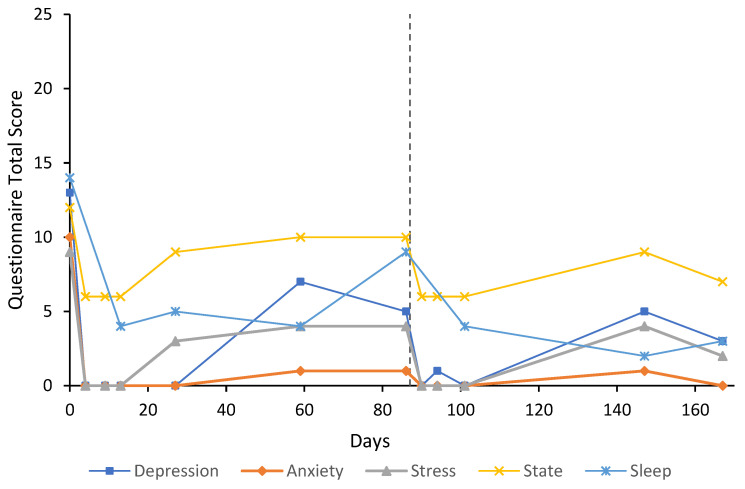
Line graph showing Case 8 results from the DASS-21 (depression, anxiety, and stress subscales), SSAI-6, and JSS at each follow-up. The participant was treated with an eight-day flumazenil infusion and re-treated on day 87 with a four-day flumazenil infusion (denoted by a dashed line).

***Case 8*** was a 50-year-old male, crane operator, with a 10-year history of GAD and an alcohol use disorder (2.5 L of wine per day). The participant was treated with a naltrexone implant for their alcohol use disorder for approximately four months, and again three weeks prior to treatment with flumazenil, and remained mostly abstinent from alcohol. The participant had been attending counselling sessions for his alcohol use. U&E, LFT, FBC, and TFT were unremarkable at baseline. Sodium valproate 500 mg twice a day was given for seizure prophylaxis over the duration of the first flumazenil treatment (eight days). Baseline anxiety and stress subscale scores were extremely severe (10) and mild (9), respectively (Figure 8). Treatment response and remission were achieved on both anxiety and stress subscales by day 4 and were maintained through to day 167. On day 86, the participant felt as though the anxiety was returning, which they partly attributed to a period of stress from a breakup with their partner reported at the follow-up on day 59. As such, they received a further four-day flumazenil infusion on day 86. Depression subscale scores remained below baseline for the entire follow-up period. Depression at baseline was scored severe; however, this ranged from normal to moderate at subsequent follow-ups. State anxiety was decreased from baseline (12) to the lowest score possible (6) for most of the follow-ups. Sleep was also improved from baseline (14), reaching a minimum of 2 by day 147. The participant continued to attend counselling sessions for his alcohol use and anxiety throughout the follow-up period. Trait anxiety at baseline was 54 and decreased to 41 by day 86. At the final follow-up on day 167, trait anxiety had decreased 22 points from baseline to 32. On day seven, blood test results (U&E, LFT, and FBC) remained unremarkable.

## 4. Discussion

The results of this case study report the long-term results of eight cases that received low-dose continuous flumazenil infusions for GAD, most with a history of treatment resistance. Although the majority of participants required re-treatment at some point during the follow-up period, Case 1 appeared to have sustained improvements over the six months with only one eight-day infusion period. Of significance, all participants had at least a 50% reduction in anxiety or stress symptoms assessed by the DASS-21 either during or after treatment with flumazenil and no abnormalities in LFT, U&E, or FBC were attributed to flumazenil from blood tests taken opportunistically. As seen in Figure 1, Figure 2, Figure 3, Figure 4, Figure 5, Figure 6, Figure 7 and Figure 8, measures of anxiety and stress tend to decrease after initial treatment and subsequent re-treatment; however, it is observed that symptoms gradually increase again over time approaching baseline levels. 

The theory of the use of flumazenil in anxiety disorders, such as GAD, postulates that the increase in allopregnanolone in response to acute stress [11,28] and subsequent decrease during chronic stress [29,30,31,32] alters GABA_A_ receptor composition; specifically, an increase in α_4_βδ GABA_A_ receptors and α_4_ expression [33,34,35]. This results in decreased inhibition [36], and the receptor expression is associated with an anxiogenic response to allopregnanolone compared to its usual anxiolytic action in animal models of different conditions [37,38]. As flumazenil has been shown to remove these receptors [12], theoretically, this would result in a normal anxiolytic response to allopregnanolone during stress. However, this means that chronic stress following flumazenil treatment would result in these altered GABA_A_ receptors repopulating and perpetuating anxiety symptoms. As allopregnanolone is one of the most potent positive allosteric modulators of the GABA_A_ receptor with anxiolytic properties [39], restoring the endogenous function of this neurosteroid could prove to be an effective treatment for anxiety. The observed pattern in the results certainly supports this theory, with an initial decrease in anxiety and stress symptoms, followed by a gradual increase as the participants are exposed to stressors. Subsequently, anxiety and stress decreased after re-treatment; in particular, Cases 4, 5, and 6, who experienced significant life events, which were inherently stressful and anxiety-inducing. 

Interestingly, treatment response and remission on the anxiety subscale occurred more quickly than on the stress subscale. This indicates that flumazenil may work more effectively on symptoms addressed in the anxiety subscale compared to the stress subscale. These symptoms tended to be more somatic in nature (i.e., dry mouth, difficulty breathing, trembling, and awareness of heartbeat), which may be due to the GABAergic involvement in the somatic manifestations of anxiety [40,41].

Based on this theory, continuous treatment with flumazenil may prevent the remitting nature of GAD rather than intermittent treatment to internalise altered GABA_A_ receptors as they repopulate. However, the pharmacokinetic profile of flumazenil makes this difficult to achieve (short half-life, low bioavailability, and extensive first-pass metabolism). A previous study using low-dose flumazenil for idiopathic hypersomnia addressed this issue by using a subcutaneous implant, which delivered approximately 0.35 mg per tablet per day with five tablets (1.75 mg/day) implanted [42]. This, however, required surgical implantation under local anaesthetic, which may not be accessible, cost-effective, or accepted by all patients. One potential alternative is a patch delivery system, which would be less invasive and require less involvement from medical staff. Caution is advised if increasing the dose or delivery rate in any new devices or delivery systems, as the dose and rate in this study (4 mg/24 h) has data to support that it is unlikely to be anxiogenic [43], unlike bolus doses. However, this data was collected during benzodiazepine withdrawal rather than GAD. 

## 5. Strengths and Limitations

There are inherent limitations associated with case series, namely the lack of a comparison group. Although the sample size is small, the study design allowed for a relatively inexpensive and long-term follow-up of participants. As a preliminary assessment of the safety and efficacy of long-term continuous low-dose flumazenil, the results provide promising evidence that warrants more research. A notable strength is that the cases represent predominately difficult-to-treat patients with psychiatric comorbidities and treatment resistance, which represents a commonly presenting population in psychiatry. Additionally, we aimed to control for the confounding effect of other medications by only including those that had not initiated or changed medication for GAD in the past six weeks. Although access to psychotherapy may have confounded results, only one participant attended sessions frequently and consistently; therefore, this was unlikely to have changed patients’ outcomes. 

## 6. Future Research

Future research should be directed at confirming these results in a pilot double-blind randomised setting, which utilises a predefined protocol for the re-treatment of participants (e.g., a cut-off point on the relevant anxiety scale). Additional research aims are to determine the utility of alternate and uninterrupted drug delivery systems and determine predictors of treatment success as this population is psychiatrically diverse. As Case 1 was the only participant to have a sustained response to flumazenil over the six-month period with one eight-day infusion, efforts could be directed to investigating the use of flumazenil on anxiety symptoms for comorbid GAD in ADHD. 

## 7. Conclusions

Low-dose continuous infusions of flumazenil were associated with treatment responses in all participants measured on the anxiety and stress subscales on the DASS-21. Anxiety measures tended to decrease during the infusion and remain low for some time; however, symptoms gradually returned. In participants who were re-treated, anxiety levels decreased again. We did not observe any remarkable changes in liver function, kidney function, or full blood count blood test results collected opportunistically. Although these results are promising, future randomised control trials are required to confirm these results. 

## Figures and Tables

**Table 1:** Summary of baseline participant characteristics. * Total dose delivered over the entire observation period. ^ Does not include current GAD drug therapy. ADHD, attention deficit hyperactivity disorder; AUD, alcohol use disorder; GAD, generalised anxiety disorder; SR, slow release.

ID	Age at First Infusion (Years)	Total Cumulative Flumazenil Dose (mg) *	Duration of GAD (Years)	Comorbid Psychiatric Conditions	Current GAD Drug Therapy (mg)	Previously Taken Medication for GAD ^	Sodium Valproate Given	Re-Treatment Day
**1**	29	32	10	ADHD	Lisdexamfetamine Agomelatine 25	Y	N	-
**2**	35	48	15	Alcohol abuse	Escitalopram 20	Y	Y	56
**3**	64	48	15	PTSD	-	Y	N	28
**4**	51	64	20	Social anxiety	-	Y	N	49
**5**	20	64	8	Social anxiety	-	Y	N	182
**6**	54	64	5	-	Bupropion SR 150 Fluoxetine 20	Y	N	90
**7**	31	32	20	Depression	-	N	N	-
**8**	50	48	10	AUD	-	N	Y	87

## Data Availability

Not application.
